# Oncology Clinicians' Perspectives of a Remote Patient Monitoring Program: Multi-Modal Case Study Approach

**DOI:** 10.2196/60585

**Published:** 2025-01-24

**Authors:** Ann Marie Mazzella-Ebstein, Robert Daly, Jennie Huang, Camila Bernal, Clare Wilhelm, Katherine S Panageas, Jessie Holland, Rori Salvaggio, Jill Ackerman, Jennifer Cracchiolo, Gilad Kuperman, Jun Mao, Aaron Begue, Margaret Barton-Burke

**Affiliations:** 1 Nursing Research, Department of Nursing, Memorial Sloan Kettering Cancer Center, New York, NY, United States; 2 Thoracic Services, Department of Medicine, Memorial Sloan Kettering Cancer Center, New York, NY, United States; 3 Memorial Sloan Kettering Cancer Center, New York, NY, United States; 4 Hospital Administration, Memorial Sloan Kettering Cancer Center, New York, NY, United States; 5 Department of Surgery, Memorial Sloan Kettering Cancer Center, New York, NY, United States; 6 Epidemiology - Biostatistics, Memorial Sloan Kettering Cancer Center, New York, NY, United States; 7 Nursing Ambulatory Services, Department of Nursing, Memorial Sloan Kettering Cancer Center, New York, NY, United States; 8 Digital Informatics & Technology Solutions (DigITs), Memorial Sloan Kettering Cancer Center, New York, NY, United States; 9 Integrative Medicine Services, Memorial Sloan Kettering Cancer Center, New York, NY, United States; 10 Hospital Administration, Advanced Practice Providers, Memorial Sloan Kettering Cancer Center, New York, NY, United States

**Keywords:** cancer, oncology, clinician end users, remote patient monitoring, digital health, implementation science, patient monitoring, patient access, care, communication, usability, functionality, survey, interview, efficiency, workflow, user, clinician support

## Abstract

**Background:**

Remote patient monitoring (RPM) aims to improve patient access to care and communication with clinical providers. Overall, understanding the usability of RPM applications and their influence on clinical care workflows is limited from the perspectives of clinician end users at a cancer center in the Northeastern United States.

**Objective:**

This study aims to explore the usability and functionality of RPM and elicit the perceptions and experiences of oncology clinicians using RPM for oncology patients after hospital discharge.

**Methods:**

The sample included 30 of 98 clinicians (31% response rate) managing at least 5 patients in the RPM program and responding to the mHealth usability between March 2021 and October 2021. Overall, clinicians responded positively to the survey. Item responses with the highest proportion of disagreement were explored further. A nested sample of 5 clinicians who responded to the study survey (30% response rate) participated in interview sessions conducted from November 2021 to February 2022, averaging 60 minutes each.

**Results:**

Survey responses highlighted that RPM was easy to use and learn and verified symptom alerts during follow-up phone calls. Areas to improve identified practice changes from reporting RPM alerts through digital portals and its influence on clinicians’ workload burden. Interview sessions revealed 3 main themes: clinician understanding and usability constraints, patient constraints, and suggestions for improving the program. Subthemes for each theme were explored, characterizing technical and functional limitations that could be addressed to enhance efficiency, workflow, and user experience.

**Conclusions:**

Clinicians support the value of RPM for improving symptom management and engaging with providers. Improvements to address RPM challenges include functional changes to enhance the program’s utility, such as input from patients about temporal changes in their symptoms and technical resources for home monitoring devices.

## Introduction

Improving the patient’s access to care and enhancing their quality of life by preventing readmission is the primary goal of posthospital care delivery, yet traditional oncology care models often lack communication and monitoring after discharge [1-5]. Consequently, re-admissions for oncology patients within 30 days of discharge for symptoms that could be mitigated with proactive, remote patient monitoring (RPM) is an opportunity for advancing oncology patient care [6-8]. Our prior research at Memorial Sloan Kettering Cancer Center (MSK) highlights the potential of RPM to facilitate transitions of care through optimized patient-provider communication and proactive engagement through digital technology [7,8]. The RPM program was designed to reduce unplanned care visits following discharge from the hospital by proactively monitoring patient status via daily questionnaires. Electronic patient-reported outcome (ePRO) assessments generated alerts for patients enrolled in the RPM that were sent to the patient’s primary oncology care clinician team. Details regarding the early development and integration of the organizational RPM project, as well as the patients recruited have been previously published [3,9-11]. However, limited research exists describing the perspectives and experiences of the clinician end users regarding how digital interventions are integrated into clinical care and the influence of technological health care models on patient care workflows.

To address this gap, our team developed this study to gain a greater understanding of the influence of RPM on clinical workflows in daily practice as part of a larger institutional initiative supporting center-wide symptom management during the transition of care from the inpatient to the outpatient setting [3,9-11]. The purpose of this study is to explore the usability of the RPM program and the perceptions and experiences of oncology clinicians using RPM to care for patients in practice after hospital discharge. The broader aim was to identify the impact, perceived usefulness ease of use, user control of the RPM, and barriers and facilitators experienced during the initial RPM implementation. The Consolidated Framework for Implementation Research [12] guided this study to query oncology clinicians, the key contributors to this management strategy, about their experiences, satisfaction, usefulness, and value of RPM.

## Methods

### Overview

The study’s design used a multi-modal case study approach, using quantitative (survey) and qualitative (interviews and focus groups) to characterize the phenomenon and context of integrating RPM into the practice setting [13-18]. This approach encourages multiple sources of evidence to understand the clinician’s perspectives about the use and function of RPM in real-world clinical practice and its influence on patient care workflows.

Initially, survey methods assessed clinicians’ responses to the overall usability, functionality, and value of RPM. Second, clinicians participated in semistructured interviews and focus group sessions to elicit feedback and explanations about items of concern found in the survey responses [13,14,17]. The interview sessions aimed to delve deeper into the survey concerns and examine the use of the RPM application from a clinical perspective.

### Setting and Clinician Sample

This study focused on clinician end users, specifically nurses involved in clinical office practices that integrated the initial organization’s RPM initiative [3,9-11] from October 15, 2018, to July 10, 2019, at MSK in the Northeastern United States. The clinicians in this study were the first group of end users involved in caring for oncology patients using RPM. The RPM program was rolled out sequentially, beginning with the oncology services that care for patients with the highest symptom burden (Table 1). Clinicians from physician office practice settings, who had experience in caring for at least 5 patients enrolled in RPM, were invited to participate in the usability survey. This criterion ensured that the clinicians had ample experience with using the RPM program. Additionally, a nested sample of clinicians who completed the survey were recruited to take part in semistructured interviews to provide further information about the challenges of the RPM identified from the survey [19-22].

**Table 1.** Description of practice clinicians by percent of the sample. Clinicians: registered nurse in practice settings; responses (n=30; response rate 31%).

Oncology specialty disease management service	m-Adapted health questionnaire disseminated, n (%)^a^
Thoracic (THR)	23 (24)
Gastro-intestinal (GI)	30 (31)
Breast (BR)	15 (15)
Sarcoma (SAR)	6 (6)
Genito-urinary (GU)	6 (6)
General medicine oncology (GMO)	5 (5)
Head & neck (HN)	4 (4)
Melanoma (MEL)	2 (2)
Myeloma (MYL)	2 (2)
Bone marrow transplant (BMT)	1 (1)
Lymphoma (LYMP)	1 (1)
Leukemia (LEU)	1 (1)
Covering clinicians/no specific service	2 (2)
Total surveys sent	98 (100)

a Percentage of the sample.

### Data Collection

#### Survey

Eligible clinicians in this study who met the study criteria were identified using the RPMs dashboard. Clinicians were recruited using the organization’s email with a link to a web-based consent and m-Adapted Health Usability Questionnaire from March 2021 to October 2021. To maintain the anonymity of the clinicians, broad demographic data were collected to quantify clinicians’ years of experience in their current organizational roles and use RPMs within electronic health record systems.

The m-Adapted Health Usability Questionnaire [23,24], is a 29-item self-report survey, that assessed an overall understanding of the functionality of the RPM from the perspectives of the clinician end users including the quality of work life, perceived usefulness, and ease of use, and user control of RPM in a 7-point Likert Scale (1 [strongly disagree] to 7 [strongly agree]). The questionnaire takes about 13 minutes to complete in REDCap (research electronic data capture; Vanderbilt University) [25]. The Cronbach α for all scale values was >0.7 with scores ranging from 0.85 to 0.92. Permission to use the questionnaire was not required [24].

#### Interview Sessions and Guide

Clinicians who completed the study’s survey were also recruited through the organization’s email to take part in 60-minute interviews or focus group sessions. The interview guide was developed by the research team based on understanding areas where the usability of RPM was challenging for the clinicians. Items from the usability survey where the percentage of responders who disagree, disagree, and strongly disagree exceeded 44%, informed the interview and focus group guide. Topics in the guide were broad enough to elicit narrative data describing the background and contextual characteristics related to the clinician’s experience with RPM [26,27]. The interview guide was organized into four topics: (1) General understanding and information about the RPM; (2) Experiences, barriers, and challenges with using the RPM; (3) The Influence of the RPM on the current workflow; and (4) Suggestions for improvement (Table 2). Interviews and focus groups were conducted via the web by the PI (AMME) and the qualitative methods specialist (MBB) from November 2021 to February 2022. Verbal consent was obtained from clinicians to record sessions.

**Table 2.** Interview guide.

Topic	Main statements	Probe questions	Aligns survey
Topic 1: provide general information about the topic of study and ask questions related to the specific service and the specific patient needs.	Where do you work? Can you tell me about your work with RPM^a^? What can you tell us/each other about RPM?	Can you briefly describe the patient group in your office practices (patient symptom burden, any factors in this group that need to be addressed through RPM)? Can you describe your workflow and the technology systems used in your daily work activities? Can you describe the workflow, and the systems used when providing care? How does the RPM fit into the care workflow?	Q^b^ 3,4
Topic 2: issues and barriers that are specific to using the RPM.	What are the strengths/barriers to using RPM from the nursing perspective? What are the strengths/barriers to using RPM from the patient’s perspective? Do you think RPM is valuable: to nurses the patients?	What functionality is found in the current systems and applications used during patient care activities to make caring for patients easy? What functionality in the RPM is not working? Describe? Do you think the patients like using the RPM? Does the RPM provide value for the patients? Care? Do you think patient education about the RPM helps?	Q 7,9,10,13
Topic 3: practice workflow; getting to the issue about the RPM, frequency of using the toolkit, and the influence of workflow.	How does the RPM toolkit work with your workflow? How do you use the RPM apps to provide care? How do you use the RPM toolkit using the digital apps?	Describe how you use the RPM (and patients with remote monitoring devised, ie, pulse oximetry) How do you use the information in caring for your patients? Can you describe the communication among practitioners using the RPM?	Q 18‐20
Topic 4: functionality, if modified, would be meaningful and helpful for the RPM, improved workflow, user satisfaction, and improved patient outcomes.	What are your thoughts about how RPM functions? How could it be improved?	If you could fix anything about the RPM, what would it be? To what extent do you think the modifications described could influence patient outcomes that patient care? What current functionality has the greatest influence on workflow?	Q 23‐25

a RPM: remote patient monitoring.

b Q: question.

### Data Analysis

#### Survey

Survey response data were extracted from REDCap [25] to a deidentified Excel spreadsheet before analysis. To ensure anonymity, demographic information was only provided for clinicians who were sent the surveys and not collected for clinicians who responded. Data responses were initially scored in REDCap [25] for the frequency and proportion of agreement or disagreement with survey items consistent with the 7-point response scale. The scale responses were then collapsed into 3 groups, disagreement (strongly disagree, disagree), neutral (neither agree nor disagree), and agreement (agree and strongly agree). The Strengthening the Reporting of Observational Studies in Epidemiology guidelines were used to report the study’s quantitative findings [28].

#### Interviews Sessions

Data from the interviews and focus groups followed the Consolidated Criteria for Reporting Qualitative Research guidelines and were used to report the study’s findings [29]. The rigor and validity of the data were supported by clarifying participants’ statements during interview sessions. The transcripts were audio recorded, transcribed verbatim, and independently reviewed by 2 reviewers using thematic content analysis [30]. Themes and subthemes were constructed based on the verbal responses from the clinicians. Team consensus was determined by agreeing upon the best representation of the data [24,26,27,30].

### Ethical Considerations

This research is part of an ongoing organizational program of RPM initiatives. This research obtained ethical approval from the MSK Institutional Review Board (X20-086) as exempt research and follows the ethical principles and guidelines of the Belmont Report. All responders to surveys completed web-based informed consent to participate. Participants in focus groups and interviews provided informed verbal consent.

## Results

### Overview

A total of 35 clinicians participated in this study from both survey and interview sessions. Of the 98 clinicians who had experiences with at least 5 patients enrolled in RPM and received the study’s survey, 30 responded (31% response rate). A nested sample of 5 clinicians (17% response rate) from this original group of 30 clinicians who completed the survey, also agreed to take part in 4 sessions (3 interviews; 1 focus group of 2 clinicians). Demographic data were not collected with the survey to maintain the anonymity of the clinician responders. However, the majority of clinicians who responded to the survey and interview sessions represented the Gastrointestinal, Thoracic, and Breast oncology services known to have the highest symptom burden. Of the clinicians who took part in the interview sessions, 3 clinicians had 5 or more years of organizational work experience, while 2 clinicians had 2 years or less of experience.

### Survey Responses

Overall, the clinicians reported that the RPM was easy to use and learn and that symptoms communicated through the RPM program were confirmed during follow-up phone calls. Suggested areas for improvement included communication and practice changes related to symptom management and the clinicians’ workload burden. Findings highlighting survey items with the highest frequency and proportion of survey responses are presented (Table 3).

**Table 3.** Frequency distribution of m-Adapted health questionnaire responses (n=30)^a^.

Statements about remote patient monitoring (RPM)		Total responses, n	Agree/somewhat agree/strongly agree, n (%)^b^	Neither agree nor disagree, n (%)	Disagree/somewhat disagree/strongly disagree, n (%)	Unknown, n
Ease of use and functionality as easy to learn						
	1. RPM is easy to use	27	18 (67)	2 (7)	7 (26)	3
	2. RPM is easy for me to learn	27	18 (67)	7 (15)	5 (18)	3
	3. Liked the digital interface of the patient data received through portal secure message alerts	26	8 (31)	4 (15)	14 (54)	4
	4. I liked the digital interface of the patient data received through the Splunk/summary dashboard	26	7 (27)	12 (46)	7 (27)	4
	5. Information in the Splunk/summary dashboard was well-organized	25	12 (48)	9 (36)	4 (16)	5
Integrating the RPM into workflows						
	6. RPM has usable functions and capabilities	27	14 (52)	6 (22)	7 (26)	3
	7. RPM has been appropriate for me to care for patients	27	11 (44)	1 (4)	14 (52)	3
	8. Easy to integrate into my current clinical workflow	27	11 (41)	4 (15)	12 (44)	3
Acceptable for practice						
	9. An acceptable way to coordinate health care services	26	9 (35)	5 (19)	12 (46)	4
	10. Improved communication between my colleague’s office practice teams for patient symptoms	26	10 (38)	1 (4)	15 (58)	4
	11. Prompts me to refer patients to a specialist for symptom management	25	9 (36)	5 (20)	11 (44)	5
	12. RPM is useful for my health care practice	26	12 (46)	4 (15)	10 (39)	4
	13. Improved my ability to deliver health care services	27	7 (26)	4 (15)	16 (59)	3
	14. Helped me manage my patient’s symptoms effectively	27	11 (41)	5 (18)	11 (41)	3
RPM convenience						
	15. Is convenient for me to communicate with patients	26	10 (39)	4 (15)	12 (46)	4
	16. Had many more opportunities to interact with patients	27	11 (41)	4 (15)	12 (44)	3
	17. Felt comfortable communicating with my patients about symptoms using portal secure messaging	26	12 (46)	1 (4)	13 (50)	4
Devices and symptom management						
	18. Highlighted the high-risk symptoms (not pulse oximetry) provided the correct corresponding severity level for the patient-reported symptoms	27	10 (37)	5 (19)	12 (44)	3
	19. Highlighted symptoms related to pulse oximetry appropriately	27	10 (37)	9 (33)	8 (30)	3
	20. Patients were appropriately identified for pulse oximeter monitoring.	27	12 (44)	7 (26)	8 (30)	3
	21. Pulse oximeter monitoring enabled me to more effectively manage my patient’s symptoms	26	10 (38)	8 (31)	8 (31)	4
	22. High-risk symptom alerts were confirmed upon communication with the patient by telephone	27	20 (74)	2 (7)	5 (19)	3
Satisfaction, value, and recommendations						
	23. Using RPM has improved my job satisfaction	27	3 (11)	9 (33)	15 (56)	3
	24. Using RPM decreased my workload	26	2 (11)	2 (8)	21 (81)	4
	25. Adds value to how I can care for my patients	27	8 (30)	7 (26)	12 (44)	3
	26. Patients reported the value of participating in RPM	27	8 (30)	7 (26)	12 (44)	3
	27. Overall, I am satisfied with the using RPM	27	9 (33)	4 (15)	14 (52)	3
	28. I would use RPM again to monitor the symptoms of patients	27	10 (37)	6 (22)	11 (41)	3
	29. I would recommend the RPM to my colleagues	26	8 (31)	5 (19)	13 (50)	4

a Number of participants who answered the survey.

b Percentage of the group responses.

### Usability

The RPM was easy for clinicians to use but was influenced by a shift in basic assumptions with patients now instructed to report symptoms through portal messages rather than calling the medical offices (14/26, 54%).

### Integrated Workflows

The proportion of responses was fairly divided between agreement and disagreement. While 14 (52%) clinicians agreed that the RPM had usable functions and capabilities, 14 (52%) clinicians disagreed that the RPM was suitable for their patient care needs.

### Acceptability in Practice

A total of 16 (59%) clinicians reported a disagreement with the notion that RPM improved their ability to care for their patients and, 12 (46%) clinicians reported that the RPM was useful in their practice.

### Convenience in Patient Care

Despite similar proportions of agreement and disagreement overall, a higher proportion of clinicians (13/26, 50%) disagreed with feeling comfortable communicating with patients about symptoms through portal messages.

### Symptom Management

Clinicians reported that devices were appropriately used for 12 (44%) patients and that high-risk symptoms were confirmed during follow-up phone calls to 20 (74%) patients. The responses were fairly split between agreement (10/27, 37%) and disagreement (12/27, 44%) on how well the devices provided severity levels. Additionally, 10 (38%) patients agreed that RPM helped them manage their patients.

### Satisfaction and Value

A total of 21 (81%) clinicians reported that the RPM did not decrease their workload and 15 (56%) clinicians reported it did not improve job satisfaction. In addition, 14 (52%) of the clinicians were not satisfied with RPM, and 13 (50%) clinicians would not recommend the RPM to colleagues. The proportion of clinicians who neither agreed nor disagreed was similar to those who reported disagreement and was further explored in the interview sessions.

### Interview Sessions

The interviews and focus group sessions consistently provided similar information about the RPM thus achieving thematic saturation [30]. All clinicians reported a limited understanding of the RPM during its implementation and suggested that ongoing educational modules and supporting technical support would enhance the RPM program for the clinician end users. Three major themes emerged from the interviews: clinician understanding and usability constraints, patient constraints, and suggestions for improvement. Subthemes were further explored (Table 4).

**Table 4.** Themes and subthemes.

Theme	Subthemes
Theme 1: Clinician understanding and usability constraints	Clinician uncertainty Repetitive alerts Alignment with clinician workflows Program value
Theme 2: Patient constraints	Appropriate patient enrollment Timing of enrollment Communication during and after enrollment
Theme 3: Suggestions for improvement	Clinical champions needed Program modifications Information technology support for end users

### Theme 1: Clinician Understanding and Usability Constraints

#### Overview

Theme 1 encompasses the information given to the clinicians during their RPM orientation and how they applied the application to their current workflow. Three subthemes included clinician uncertainty, repetitive alerts, alignment with clinician workflows, and program value.

#### Subtheme: Clinician Uncertainty

The RPM was rolled out in stages starting with oncology services known to have patients with high symptom burdens. The clinicians involved in the initial rollout reported more knowledge and understanding about the RPM compared with clinicians who were involved later in the rollout. Clinicians joining later in the rollout reported having little training about the RPM. However, they reported that the increased frequency of using the RPM helped them navigate the program for addressing symptom management alerts and functionality.

*I have only worked with it (RPM) for a couple of patients in the outpatient setting, but I get a notification (through) the portal … to notify us that the patient has enrolled in the RPM program…During this timeframe patients (complete) a survey every day about their symptoms and how they feel.*

[R32]

#### Subtheme: Repetitive Alerts

All clinicians reported concerns about repetitive alerts from daily patient surveys. Each day that a patient reported a symptom and generated an alert [1,3,10], clinicians called the patient to verify it and confirm if the symptom was worse, better, or the same as the previous survey. Although they could see past patient response trends in the system, this required substantial effort and did improve the patient’s care for symptoms. When alerts were consistently reported, the clinicians used their judgment to decide when to call patients for verification.

*There are a couple of categories that we feel were a little monotonous. ie, was (the pain/symptom) worse? If they are at stage four lung cancer, (the patient) is on treatment and they have fatigue, every day… (the patient cannot modify) their answers, (for example) moderate fatigue; I am having trouble doing my activities of daily living, so they are clicking that every day.*

[R11]

#### Subtheme: Aligning With Clinician Workflow

Clinicians reported that before implementing the RPM, patients were instructed to call the physician’s medical offices to report symptoms. Although none of the clinicians reported significant changes to their clinical workflows, they all raised concerns about the shift from patients calling the medical oncology office for unrelieved symptoms through a digital portal. This change made clinicians apprehensive about potentially missing symptoms reported in patient portals.

*Before RPM, patients were not supposed to report symptoms through the portal, but a lot of patients, ended up just doing that because they were home.*

[R11]

#### Subtheme: Program Value

Clinicians reported that the RPM was of immense value to them and their patients and supported its inclusion in the organization’s future care delivery. Patients liked that their office practice clinician proactively contacted and interacted with them after they were discharged from the hospital.

*I do not think they (the patients) mind and love to be followed up closely, I mean my patients would love for me, a call them every day. I think patients prefer (clinicians) calling them over calling the office.*

[R41]

### Theme 2: Patient Constraints

#### Overview

The patient constraint themes involved the transition of care from the inpatient setting to RPM following discharge. Three subthemes emerged: the appropriate patient enrollment, the timing of enrollment, and communication during and after enrollment.

#### Subtheme: Appropriate Patient Enrollment

Nurses from the discharging inpatient unit were responsible for educating patients about using RPM and program enrollment. However, office practice clinicians stated that not all patients enrolled by the discharge team were appropriate due to a lack of technical proficiency, ability, and understanding of the purpose of the RPM. Clinicians consistently reported that they should be included in RPM enrollment decisions for their patients.

*There is a disconnect, (between enrolling the patient and educating them about the program) and sending symptoms via the portal messaging using the patient portal. The policy is that we are given two business days to answer portal messages…clearly, we cannot do that when it is a separate message. We need to speak to that person (By phone).*

[R21]

#### Subtheme: Timing of Enrollment

Office practice clinicians reported that the time of discharge is overwhelming for patients and inpatient unit teams have limited time to prepare the patients to leave the hospital. During this study, pandemic-related social distancing restrictions prevented caregivers and family members from taking part in the discharge process, further complicated RPM enrollment.

*The second thing that I wanted to suggest for patients wanting to quickly leave the hospital and be discharged and suddenly just get bombarded with all this information, is to discuss the RPM program the day (before) discharge.*

[R32]

#### Subtheme: Communication During and After Enrollment

Clinicians reported that a patient’s age was not a factor in RPM enrollment, as older adults familiar with technology could engage digitally with providers. However, patients with language barriers or limited access to electronic devices needed assistance from caregivers or family members similar to findings in a prior study [8]. The RPM assessments were intended to be completed during weekday work hours, from 9 AM to 5 PM Monday through Friday. Clinicians described receiving the surveys late in the day, or after hours added stress for the nursing team to address alerts.

*We have a lot of foreign-speaking patients ---, you know it is not them who are filling out the survey ... and it may be one of their relatives or their daughter. But the daughter may not live with them ... so how do you know what is going on?*

[R11]

### Theme 3: Suggestions for Improvement

#### Overview

The Clinicians supported using RPM to sustain patient care posthospital discharge. From their perspective, 3 subthemes emerged, the need for clinical champions, technology support for end users (clinicians and patients), and specific RPM modifications.

#### Subtheme: Clinical Champions Needed

Clinicians expressed the need for continuing education and updates on RPM improvements, infrequent use required relearning the system for each new patient. While there were few Clinical Champions during the initial rollout, additional experts would have helped them navigate the program and supported novice clinicians. They also suggested developing informative slides or videos addressing specific program issues.

*I think nursing (clinician end-user) … needs more education on the portal because it (the portal) is such a big part of our job. More training is needed for using (connecting) pulse ox to an iPhone (or other devices ie, Android).*

[R11]

#### Subtheme: Program Modifications

Clinicians reported that patient enrollment before hospitalization or early in their hospital admission would improve the transition from inpatient to outpatient care. They also requested allowing patients to change or clarify their symptom responses within the assessment. Frequent patient alerts were seen as potentially problematic [31] and suggested that enabling patients the option to modify their assessment responses would provide more accurate symptom information to the clinical team.

*There should be a way for them (patients) to say (respond in the survey) no changes or something… so that we don’t get the same exact thing (response) that we spoke to the patient yesterday---about because it’s not realistic, that I call the patient every day to talk to them about the same time (symptom).*

[R42]

#### Subtheme: Information Technology Support for End Users

Clinicians raised concerns about the lack of integrated IT systems they use for delivering patient care. Additionally, clinicians expressed the need for greater IT support for both patients and staff. Clinicians conveyed concern about the difficulty in resolving connectivity issues and requested dedicated assistance from IT teams.

*The patients are calling in a panic and you are trying to walk them through over the phone how to set up the device (RPM on patient’s device ie, phone, tablet). If it was not set up correctly on discharge, or the device is just not working. (These issues) add more stress to the patient, but it also adds stress to you (the clinician) This becomes the added work.*

[R11]

## Discussion

### Principal Results

This study presented the perceptions and experiences of oncology clinicians when caring for their patients using RPM after hospital discharge. Initially, clinician survey responses highlighted concerns about their understanding, perceptions, and challenges when using RPM as well as its influence on their clinical workflows. These topics were further explored in interview sessions and revealed 3 themes: clinician understanding and usability constraints, patient constraints, and suggestions for improvement. Most clinicians found the RPM easy to use and learn, allowing them to confirm the patient’s reported symptoms during follow-up phone calls. They supported RPM’s value for both clinicians and patients as a care delivery method in oncology practice, aligning with findings from other organizational studies using ePROs [11,31-36], as well as the challenges and limitations of addressing symptom alerts after the patient transitions from an inpatient to the home setting [7,35,36].

### Challenges and Opportunities From the Clinical Setting

This research further emphasized multiple challenges faced by clinicians during the initial RPM roll-out. A major concern was the practice change in clinical workflows whereby patients reported symptoms through electronic portals rather than contacting the medical offices by phone. Despite this change, clinicians felt their comfort level would improve with more experience, and as RPMs aligned with their workflows [7]. An opportunity to improve clinician confidence includes continuing educational tutorials from the initial RPM orientation and more technical and clinical support for both patients and clinicians. These efforts would facilitate aligning clinician workflows with clinical practice, thus leading to reduced stress and improved job satisfaction when caring for oncology patients using RPM [7].

Another challenge was patient constraints and their lack of understanding of RPM, which clinicians suggested might contribute to their uncertainty in digital symptom management reporting. Some patients completed surveys late in the clinic hours making it difficult to address symptoms. In many instances, clinicians reported that family members completed the surveys instead of the patients which raised concerns about the integrity of the symptom alerts. Further research could enhance the understanding of logistical limitations involving patients and caregivers with completing postdischarge symptom assessments at home. A similar study recommended that concerns revealed by patients and caregivers postdischarge could be included in future clinical outcomes [37]. Additionally, the timeframe for enrolling patients in RPM was another constraint identified by clinicians. Although older patients were comfortable using the RPM [38,39], enrollment at discharge was considered suboptimal. They suggested educating patients in a relaxed environment or office practices before hospitalizations would benefit patients and improve the enrollment process.

A major opportunity for RPM improvement involves addressing repetitive alerts from the daily ePRO assessments, also identified in other ePRO studies [23]. Clinicians verify all patient-reported symptom alerts. When prior symptoms are stable, these alerts could be averted to reduce their workflow burden [40,41]. To enhance effective communication with the patients, clinicians recommended adding context about symptom changes relative to the prior day’s ePRO assessment to improve the integrity of alerts.

The perspectives of all clinicians involved in this study expressed a need for additional resources to improve the effectiveness of RPM and their ability to clinically support it. The RPM was initially implemented using a core team of designated clinicians for addressing patient alerts. Studies of RPM cited the inclusion of core teams for this purpose which may be a preferred approach for clinicians in other studies [42]. However, clinicians in this research preferred a hybrid RPM program with primary care clinicians in the clinical practices addressing alerts on weekdays and centralized after-hours and on weekends. Clinicians proposed that leaders provide consistent status updates and education about RPM functionality through tutorial videos in an accessible location. Additional technological support could improve data collection and relieve clinicians of the burden of providing technical support for patients at home [41,42], which is not the best use of their skills [43].

### Strengths and Limitations

The consistent reports from survey responses and interviewed clinicians strengthen the study’s findings and provide pragmatic recommendations for workflow redesign and enhancing access to care for oncology patients postdischarge [1,2,7,31], As a result of this study, selected modifications were implemented into practice to improve the program’s experience for both clinicians and patients. These results provide important foundational work for future pragmatic trials and implementation science to enhance the usability and value of RPM in oncology practice.

The sample was limited to only clinicians who had experiences with at least 5 patients enrolled in the RPM, but typical for case studies [13-18]. They expressed strong positive or negative experiences with the RPM which may have created a selection bias. The study’s design was multi-modal which could have included both interviewer and report biases. Clinicians were also employees of the organization and therefore specific demographics were not collected to ensure their anonymity. This study was conducted at one comprehensive cancer center, limiting the generalizability of results. However, the perspectives of clinician end users from oncology services with known symptom burdens can apply to similar RPM implementation initiatives in other organizations worldwide.

### Conclusion

The National Cancer Institute calls for studies about cancer-related interventions, which also include program effectiveness from the perspectives of both patients and clinician end users [44]. This study contributes to the National Cancer Institute’s initiatives, demonstrating that RPM is a valuable method for communicating with clinical providers and managing patient symptoms during transitions of care from inpatient to the home setting.

## Abbreviations

ePRO: electronic patient-reported outcome
MSK: Memorial Sloan Kettering Cancer Center
REDCap: research electronic data capture
RPM: remote patient monitoring
